# A filarial parasite potentially associated with the health burden on domestic chickens in Japan

**DOI:** 10.1038/s41598-024-55284-2

**Published:** 2024-03-15

**Authors:** Naoki Hayashi, Kumiko Hosokawa, Yu Yamamoto, Sachiko Kodama, Aoi Kurokawa, Ryo Nakao, Nariaki Nonaka

**Affiliations:** 1 Laboratory of Parasitology, Department of Disease Control, Faculty of Veterinary Medicine, Graduate School of Infectious Diseases, Hokkaido University, N 18 W 9, Kita-ku, Sapporo, 060-0818 Japan; 2 Western Center for Livestock Hygiene Service, Higashihiroshima, Hiroshima Prefecture Japan; 3 National Institute of Animal Health, National Agriculture and Food Research Organization, 3-1-5 Kannondai, Tsukuba, Ibaraki 305-0856 Japan

**Keywords:** Avian filariasis, Backyard poultry, Filarial nematode, Mitochondrial genome, Phylogenetic analysis, Parasitology, Entomology

## Abstract

Chickens in free-range environments are at risk of exposure to various pathogens, such as filarioids transmitted via hematophagous vectors. However, the study of filarioids in poultry has been largely neglected compared to the extensive studies focused on viruses, bacteria, and protozoa. Here, we performed histological and molecular investigations of the filarioids detected in domestic chickens from two different flocks in Hiroshima Prefecture, Japan. In the first case, adult worms were present in the pulmonary artery and right ventricle, and microfilariae were present in multiple organs of deceased chickens. In the second case, similar filarioids were detected in the organs and blood of one necropsied layer. Phylogenetic analysis using 18S rRNA gene fragments positioned the filarioid in the same clade as that of Onchocercidae sp., previously identified in a deceased chicken from Chiba Prefecture, Japan, that is located 500 km away from Hiroshima Prefecture. Based on 28S rRNA and mitochondrial *COI* gene fragments, the filarioid was positioned distinctly from previously reported genera of avian filarioids. These results suggest that the filarioids are potentially associated with the health burden on domestic chickens and belong to the genus *Paronchocerca*. Furthermore, we developed a nested PCR assay targeting mitochondrial *COI* and detected the parasite DNA from the biting midge *Culicoides arakawae* captured near the flock, suggesting that it serves as a vector. Our findings fill the knowledge gap regarding avian filarioids, laying the groundwork for future studies examining the epidemiology, life cycle, and species diversity of this neglected parasite group.

## Introduction

Free-range chicken farming that includes backyard poultry systems is a style of poultry farming in which chickens are reared in a system with access to the outside^1^. As reported previously, this rearing style potentially provides several benefits to farmers, particularly with respect to enhancing product quality^2,3^. Its popularity is also increasing in many countries based on growing consumer concerns and the demand for animal welfare^4,5^. In Japan, although the adoption of free-range methods for commercial purposes remains limited, approximately 40 native chicken breeds are reared as backyard poultry. These native chickens serve an ornamental purpose, and 17 of these breeds are designated as “National Natural Monuments” due to their morphological and genetic specificity^6^. Despite these commercial advantages and the use of genetically important breeds, free-range chickens are easily exposed to various pathogens that can lead to a loss of health and production^7–9^.

Filariasis is a parasitic disease caused by filarial nematodes that typically undergo a life cycle involving a wide range of vertebrates, including birds, mammals, amphibians, and reptiles, as definitive hosts and hematophagous arthropods as intermediate hosts (commonly referred to as vectors)^10^. Adult female worms in definitive hosts excrete microfilariae (the first-stage larvae) that develop into infective third-stage larvae after the ingestion of vector arthropods. Third-stage larvae then invade definitive hosts via skin wounds made by arthropods, eventually developing into adult worms and producing new microfilariae^11^. Certain filarioids are known to cause severe diseases in their hosts^12,13^. The filarioids that parasitize birds belong to the family Onchocercidae and are divided into four subfamilies that include Dirofilariinae, Onchocercinae, Splendidofilariinae, and Lemdaninae^14^. With over 160 valid species in 16 genera^14^, filarioids have been reported in numerous avian species of various orders. However, studies examining filarioids in domestic poultry remain scarce^15^, and this has resulted in a lack of understanding regarding the species, pathogenicity, and epidemiology of these parasites. Characterizing such filarioids would contribute to improved management of backyard poultry, ultimately promoting increased productivity and conservation of invaluable breeds.

DNA information is valuable for diagnosis, species identification, and providing a deeper understanding of the epidemiology and phylogenetic relationships of parasites^16^. Mitochondrial DNA is particularly advantageous for some of these purposes compared to nuclear DNA, due to its higher copy number and more rapid evolutionary speed^17^. Several studies employing mitochondrial DNA have reported the successful molecular detection and characterization of protozoan parasites such as *Leucocytozoon* spp. and *Plasmodium* spp. in the peripheral blood of chickens, thus providing new insights into control strategies for parasitic diseases^18,19^. Despite these advantages, to the best of our knowledge, only a single nuclear 18S rRNA gene sequence is available for filarioids parasitizing poultry^15^. The limited availability of such DNA information poses a significant challenge to the development of molecular approaches for understanding and controlling filariasis in poultry.

Here, we describe cases of filariasis identified in free-range chickens (Chabo chickens, a native Japanese breed of *Gallus gallus domesticus*, and one Hy-Line brown layer) from Hiroshima Prefecture, Japan. To gain insight into the nature of the filarial parasites, we performed both histological and molecular investigations. Histological examination revealed that adult worms resided in the heart chamber, whereas microfilariae were present in multiple organs within the hosts. Additionally, we investigated the phylogenetic position, potential vectors, and epidemiological distribution of filarioids using nuclear and mitochondrial DNA markers.

## Results

### Case histories

#### Case 1

The location of the farm in Hiroshima Prefecture is shown in Supplementary Fig. S1. The flock contained 18 chickens comprising four breeds that include 11 Chabo, four Nagoya Cochin, two Hy-Line Brown, and one Silkie. These chickens were born in a hen house with no introductions of new chickens from the outside in the past three years, no vaccination or medical history, and no disease outbreaks. The owner fed the poultry with commercial feed and old rice and allowed the chickens to roam freely in the garden of the residence owner during the day and in a poultry house separated by breed at night. This location is surrounded by fields, a small river, and wooded areas.

On 20 July 2021, the poultry owner observed a decrease in the feed intake of the chickens, and on 26 July, the owner found that two chickens (Hy-Line Brown and Chabo) died. Three more Chabos (Chabo1–3) were found deceased the following day. The three chickens were submitted to the Western Center for Livestock Hygiene Service, Hiroshima, for further examination. Chabo1, a female, weighed 300 g and exhibited signs of emaciation. In contrast, the other two male Chabos weighed 600–700 g. Upon necropsy, Chabo2 possessed multiple white spots on the surface of the liver, whereas only one white spot was observed in the livers of other Chabos. No other abnormalities were found.

#### Case 2

The second case was identified on a chicken farm situated approximately 30 km away from the flock described in Case 1 (Supplementary Fig. S1), and there was no relationship between the two flocks. The farm housed approximately 300 chickens consisting of 100 adults and 200 chicks that were reared in a cage-free style specifically for egg laying. While the chicken coop did not offer free access to the outside for the layers, it was only separated by a net, allowing for considerable interaction with the outside environment, which was almost identical to free-range farming conditions. The owner provided the chickens with home-mixed feed, and the environmental conditions surrounding the farm were comparable to those in Case 1.

In December 2022, one to two chickens in the flock that had been introduced to the farm in October of 2022 began to die on a daily basis. In March of 2023, three debilitated chickens were subjected to pathological examination. One was a 3-year-old Hy-Line Brown layer (HLB1), whereas the other two were Araucana (AR1) and a commercial breed from a cross between Plymouth Rock and Rhode Island Red (CB1) that were born in mid-October of 2022. We observed edema and pus-like accumulation in the eyelids of poultry upon necropsy.

### Bacteriological/virological examinations

*Escherichia coli*, *Klebsiella pneumoniae*, *Bacillus cereus*, and *Staphylococcus hyicus* were isolated from the plates in Case 1, whereas *S*. *schleiferi* and *Pasteurella* sp. were isolated in Case 2 (Supplementary Table S1). The ESPLINE® Influenza A & B-N assay results were negative for highly pathogenic avian influenza. No viruses were detected in the embryonated eggs.

### Histological examination

**Figure 1:**
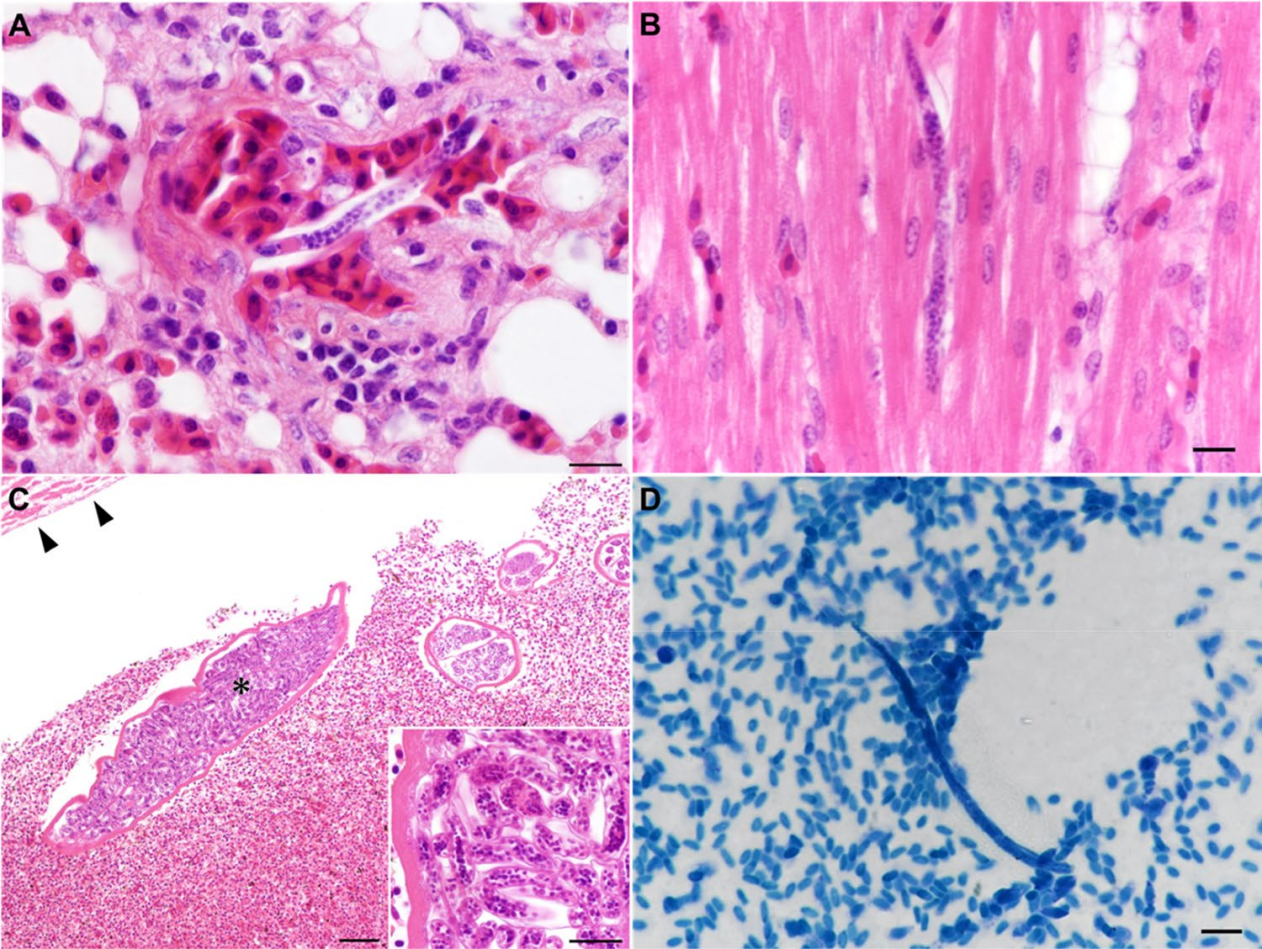
Microscopic appearances of Onchocercidae sp. detected in Chabos (Japanese Bantams) and a Hy-line Brown layer. (**A**) Histological section of a microfilaria in a small pulmonary vein of a Chabo. Hematoxylin and eosin (H&E) stain. Scale bar = 10 µm. (**B**) Microfilaria in the myocardium of a Hy-Line Brown layer. H&E. Scale bar = 10 µm. (**C**) Adult worms in a right ventricle of a Chabo. Arrowheads indicate the endocardium. The inset presents the adult worm marked with the asterisk, where many microfilariae were observed. H&E. Scale bar = 50 µm. (**D**) Microfilaria retrieved from a blood sample of a Chabo. Methylene blue stain. Scale bar = 10 µm.

Histological examination revealed the presence of numerous microfilariae in the pulmonary capillaries, pulmonary arteries, and veins of Chabos2–3 and HLB1 (Fig. 1A). Chabo1 possessed a small number of microfilariae in the lungs. In the lungs of Chabo2, scattered small granulomas contained giant foreign body cells within the parenchyma. Additionally, microfilariae were observed in the liver, kidney, and hearts of Chabos2–3 and HLB1 (Fig. 1B). Histological examination of the heart of Chabo3 revealed that a few adult worms harbouring microfilariae in the uterus were present in the pulmonary artery and right ventricle (Fig. 1C). Microfilaria concentration assays performed on blood samples recovered microfilariae measuring approximately 100 µm in length and 4 µm in width from two out of seven Chabos and from HLB1 (Fig. 1D). The tail was sharply pointed or tapering rounded, while the shape of anterior extremity was rounded.

For the Chabos in Case 1, no notable pathological changes with the exception of obstruction of blood vessels with endarteritis in the lungs of Chabo3 (Supplementary Fig. S2) were observed. In Case 2, hyperkeratosis accompanied by squamous metaplasia was observed in the esophageal glands, and clusters of lymphocytes and macrophages were observed in proximity to the glands exhibiting metaplasia. Additionally, in some glandular lumens exhibiting metaplasia, the infiltration of heterophils and macrophages and also the accumulation of cellular debris was observed.

### Phylogenetic analysis using nuclear DNA markers

**Figure 2:**
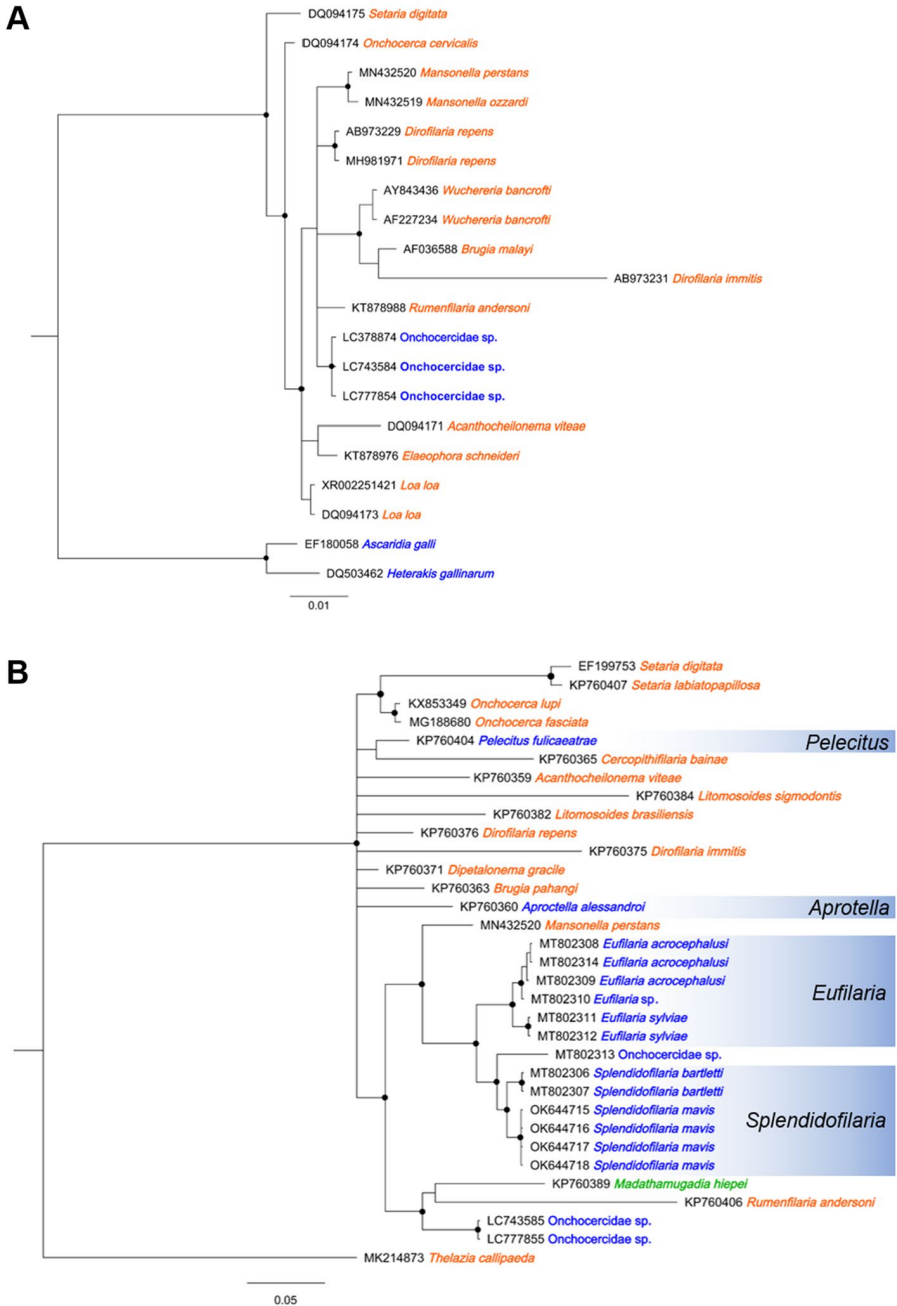
Bayesian phylogenetic trees constructed using (**A**) the nuclear 18S rRNA and (**B**) the nuclear 28S rRNA gene sequences of nematodes. Blue indicates that the parasite has avian hosts, orange indicates mammalian hosts. Black dots indicate nodes with posterior probability of > 0.75.

PCR and Sanger sequencing of nuclear 18S rRNA gene (Supplementary Table S2) were successful on DNA extracted from the lungs of Chabos1–3 and the kidney and heart of Chabo2 and resulted in nucleotide sequences of 960 bp (GenBank accession no. LC743584). The sequences from each Chabo were completely identical to each other with a high identity of 99.9% to that previously reported for a brown-colored chicken in Chiba, Japan (accession no. LC378874)^15^. Bayesian phylogenetic inference indicated that the two sequences belong to the same clade (Fig. 2A).

For the nuclear 28S rRNA gene, the sequence was successfully amplified and sequenced from DNA extracted from the lungs of Chabo2 (accession no. LC743585). Phylogenetic analysis revealed that the filarial nematode detected in Chabo2 was positioned in a separate branch from other nematodes parasitizing birds such as *Eufilaria species* and *Splendidfilaria bartletti* (Fig. 2B).

### Mitochondrial gene content and organization

**Figure 3:**
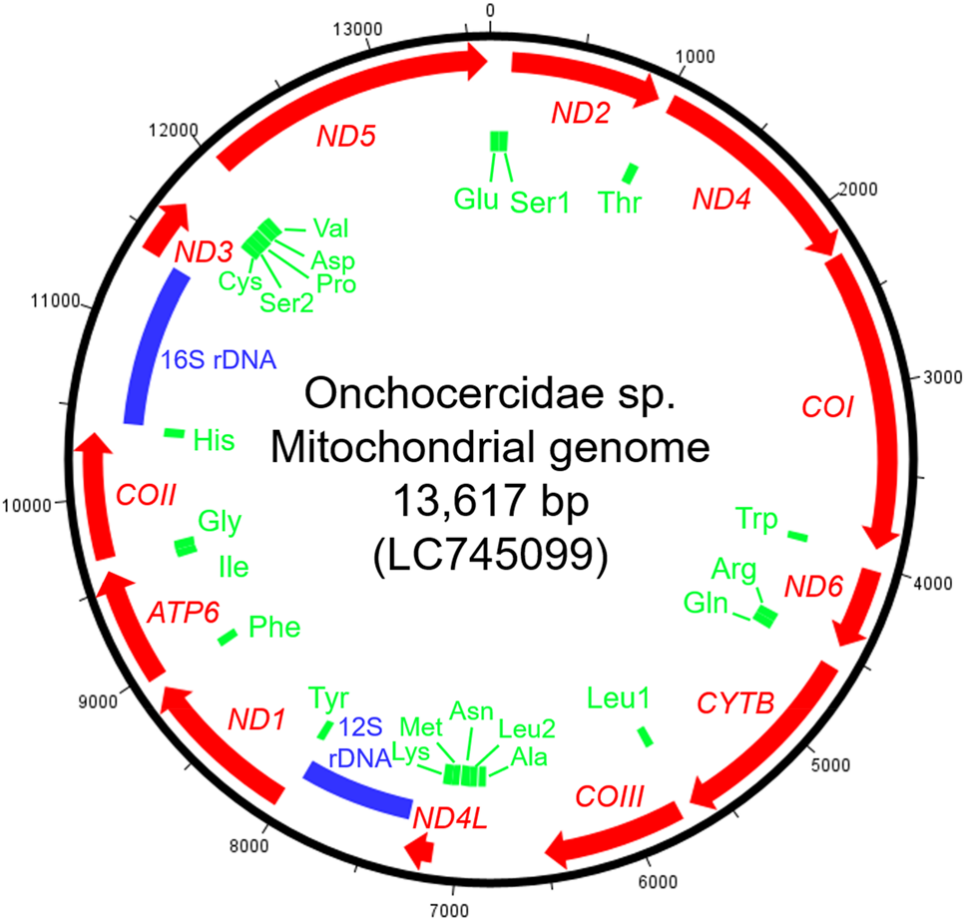
Schematic diagram of the Onchocercidae sp. mitochondrial genome. The positions of 12 protein-coding sequences (red), 2 ribosomal RNA genes (blue), and 22 transfer RNA genes (green) are illustrated.

We constructed the mitogenome of the filaria, which was 13,617 bp in length (accession no. LC745099) by an amplicon-based method (Supplementary Fig. S3). The mitogenome encoded 12 protein-coding sequences, two rDNA, and 22 tRNA genes (Fig. 3; Supplementary Table S3), and this was consistent with the mitogenomes of most nematodes. The size and AT content (79.2%) were comparable to the mitogenomes of 10 other filarial species, ranging from 13,474 to 13,839 bp in size and 73.7% to 77.7% in AT content^20,21^. The gene order was identical to that of eight other filarial species with the exception of *Chandlerella quiscali* (HM773029) that exhibited a rearrangement of tRNA gene positions^21^.

### Phylogenetic analysis of mitochondrial genes (*COI*)

**Figure 4:**
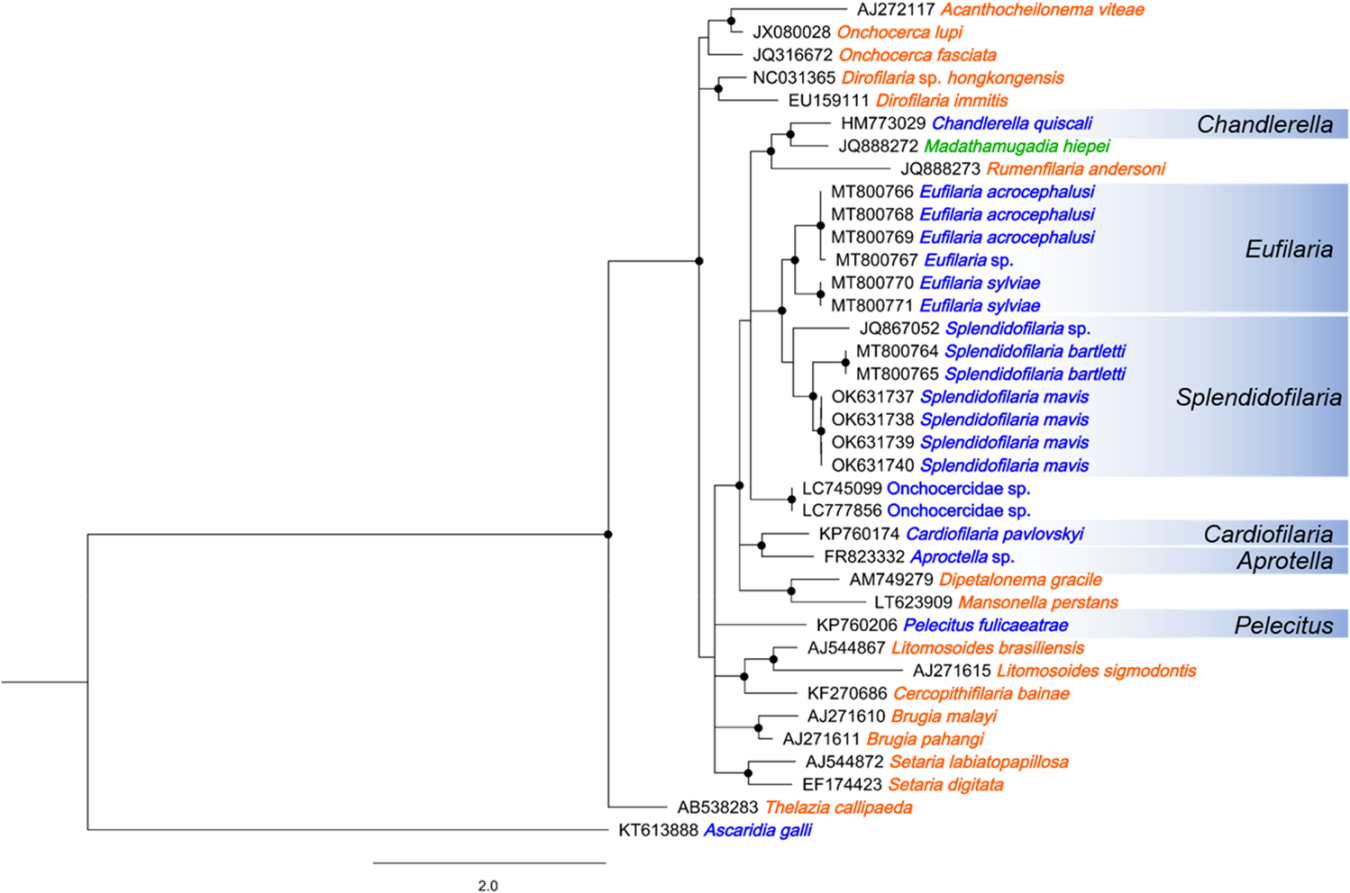
Bayesian phylogenetic tree constructed using the mitochondrial *COI* gene sequences. Blue indicates that the parasite has avian hosts, orange indicates mammalian hosts, and green indicates reptilian hosts. Black dots indicate nodes with posterior probability of > 0.75.

We utilized the mitochondrial *COI* sequence for the phylogenetic analysis, as the nucleotide sequences of a variety of filarial species are available in the database. The results were consistent with those using the 28S rRNA gene sequences, with the filaria from Chabo2 being positioned separately from other filarial species of birds (Fig. 4) and possessing nucleotide identities of 82.8–90.2% (Supplementary Table S4).

### Molecular detection of filarial DNA using the nested PCR assay in midges, blood, and organs

**Figure 5:**
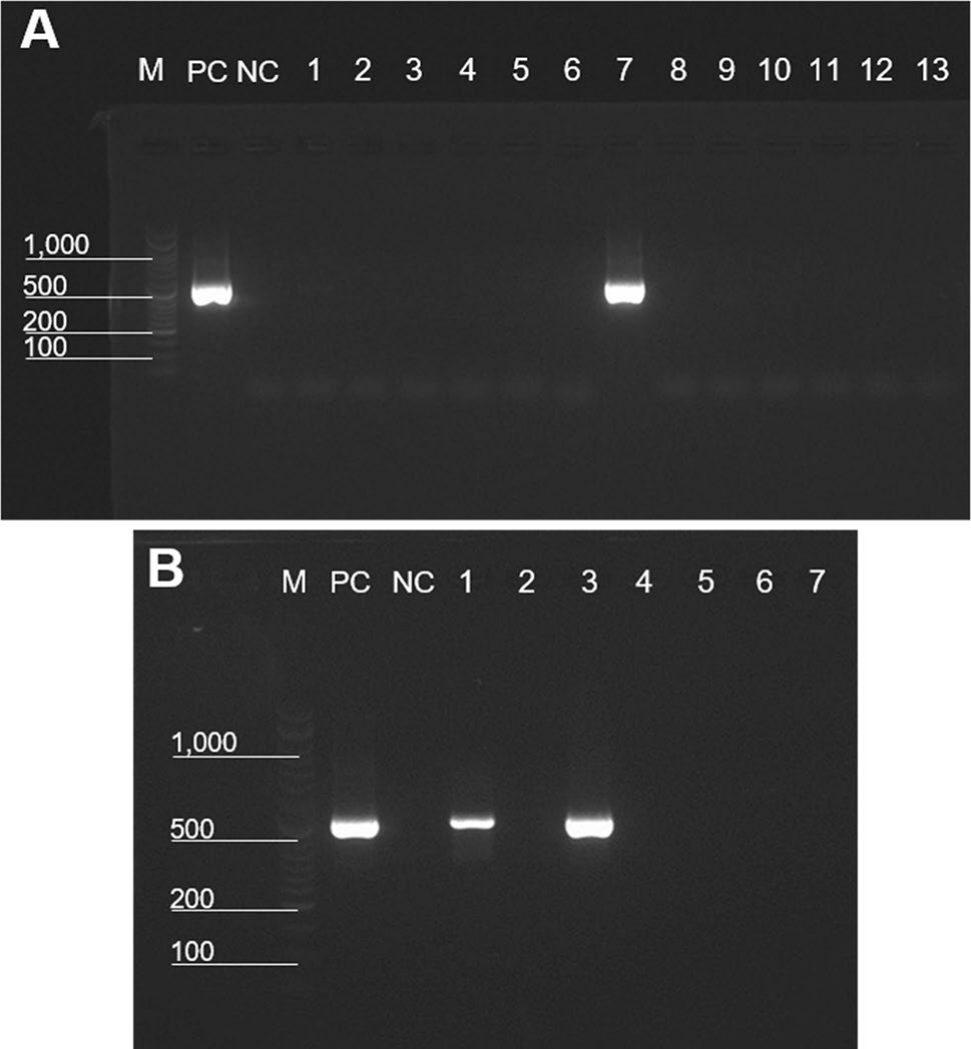
Molecular detection of Onchocercidae sp. from (**A**) *Culicoides arakawae* and (**B**) blood of Chabos that were housed with the dead Chabos using the nested PCR assay. The PCR products were analyzed in 2.0% agarose gels, and representative images are presented. (**A**) Lane M, DNA ladder; lane PC, positive control; lane NC, negative control; lanes 1–13, DNA extracted from *C. arakawae*. (**B**) Lanes 1–7, DNA extracted from the blood samples of Chabos. The original gel images are presented in Supplementary Fig. S5.

PCR targeting a 475 bp segment of the *COI* gene of *C. arakawae* yielded a single band in 48 of the 50 midge samples. The nucleotide sequences were divided into 10 haplotypes, and all of them exhibited the highest identity with *C. arakawae* in Japan (accession no. AB360975) (ranging from 98.7 to 100%), whereas the nucleotide identities with other *Culicoides* species were less than 94.2% (data not shown). Phylogenetic analysis revealed that the midges clustered with *C*. *arakawae* (Supplementary Fig. S4). The nested PCR assay successfully detected parasite DNA in six out of 48 midges (Fig. 5A and Supplementary Fig. S5). The six sequences were found to be completely identical to the *COI* sequence of the constructed mitogenome, with the exception of one sequence with a single nucleotide substitution (T–C) at position 3084 bp on the mitogenome.

Parasite DNA was amplified from blood samples collected from two of the seven Chabos (Fig. 5B and Supplementary Fig. S5) in which microfilariae were observed by microfilaria concentration (Fig. 1D). Additionally, we detected filarial DNA from multiple organs of Chabo1–3 and HLB1 using the nested PCR (Supplementary Fig. S6). All sequences derived from blood and organs were in complete concordance with the *COI* sequences of the assembled mitogenome.

### Supergroup classification of *Wolbachia* sp.

**Figure 6:**
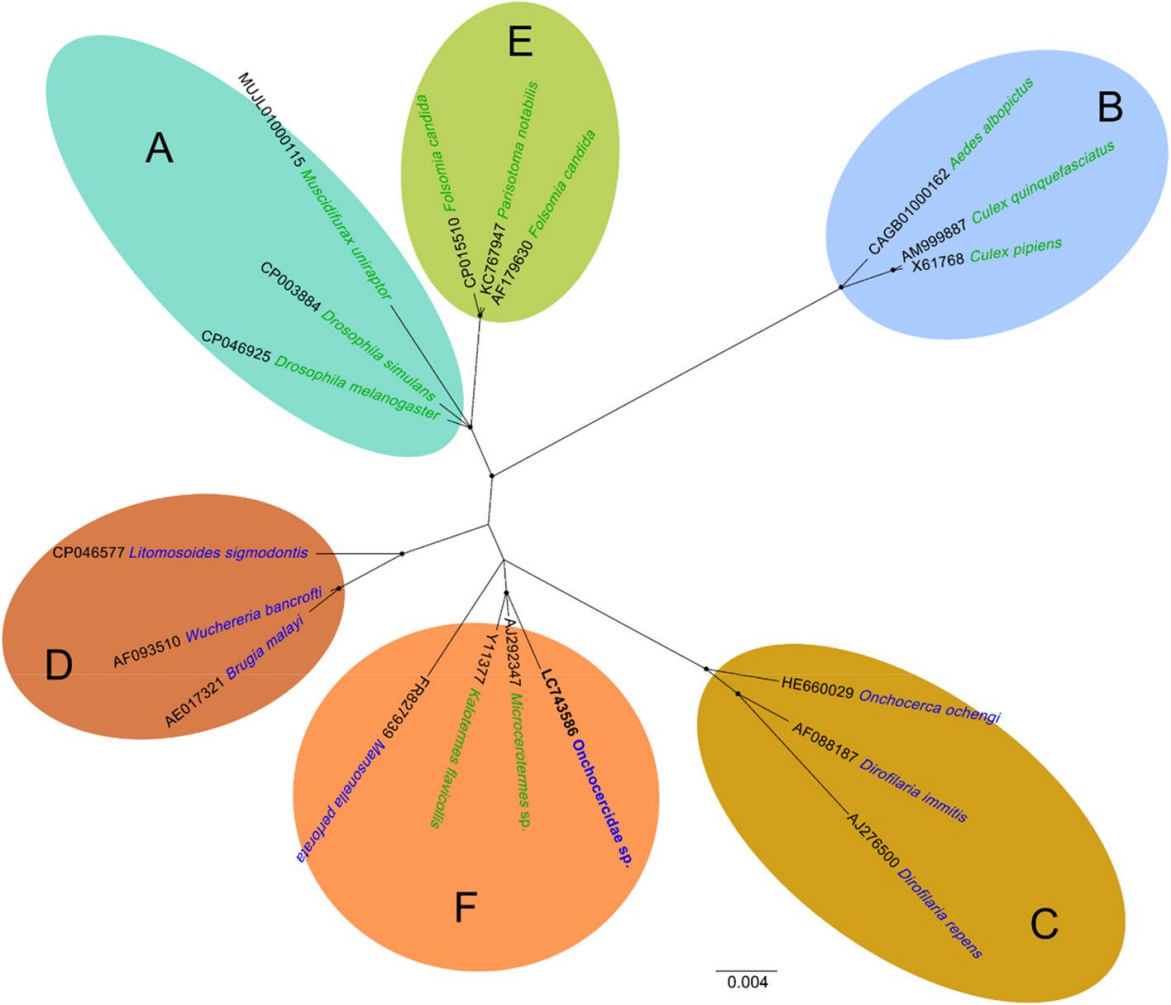
Unrooted Bayesian phylogenetic tree illustrating the relationships among *Wolbachia* detected in terrestrial arthropods and filarial nematodes. The analysis employed 821 bp of partial 16S rDNA sequences. Nodes with posterior probabilities exceeding 0.75 are denoted by black dots. Tip labels display GenBank accession numbers and their host, with green indicating arthropod hosts and blue indicating filarial nematode hosts. Supergroup classifications, as determined by previous research, are given in capital letters in the coloured circles.

The PCR successfully amplified the partial 16S rDNA of *Wolbachia* sp. (accession no. LC743586) from the lung DNA of Chabo2. The unrooted phylogenetic tree revealed that the sequence clustered with *Wolbachia* spp. of supergroup F that are present in both arthropod and filarial nematode hosts (Fig. 6).

## Discussion

Increasing the knowledge of pathogens affecting domestic chickens is a prerequisite for proper health management measures in poultry flocks. However, less attention has been paid to filarioids than has been to viruses, bacteria, or protozoa that are relevant to poultry^22^. Consequently, previous studies have been unable to identify the species or vectors of filarial nematodes (microfilariae) in backyard poultry^15,23,24^ despite their potential burden on poultry health. The aim of this study was to address this knowledge gap and provide a better understanding of filarioids in free-range chickens through morphological and molecular investigations.

Prior to this study, one study reported a case of avian filariasis in a deceased backyard chicken (with an unspecified breed) in Chiba Prefecture, Japan, and the authors deposited the 18S rRNA gene sequence of the agent in the database^15^. The filarioids detected in cases 1 and 2 possessed 18S rRNA gene sequences that were nearly identical to that of Onchocercidae sp. reported in Chiba. Phylogenetic analysis of the 18S rRNA gene sequences further confirmed that these filarioids formed a well-supported clade (Fig. 2A). These results suggest that the filarioids detected in this study and the Onchocercidae sp. reported from Chiba are likely to be the same species. As there were no introductions of domestic chickens from other flocks over the course of a year in the case in Chiba and Case 1, it is possible that the filarioid species are endemic in these areas that are located more than 500 km away from each other, thus indicating the filariasis could be prevalent over wide areas of Japan.

Morphological observations of adult worms, which are generally employed for conclusive species identification and description, could not be achieved in the current study. However, the present study identified the adult worms in the heart chamber of a Chabo chicken. Furthermore, we successfully analyzed the detailed phylogenetic relationships using both nuclear and mitochondrial DNA markers, ultimately revealing that the parasite holds a unique position. These findings allow us to infer the genus of the avian filarioid^14,25^. To date, eight of the 16 valid avian filarioid genera that include *Pelecitus*, *Paronchocerca*, *Aproctella*, *Cardiofilaria*, *Chandorerella*, *Splendidofilaria*, *Lemdana*, and *Eufilaria* have been reported to parasitize birds from the Phasianidae family, including domestic chickens^14^. Our phylogenetic analysis revealed that the filarioid detected in the Chabo was distinct from six genera previously reported with DNA sequences (highlighted in blue in Figs. 2B and 4), indicating that the parasite belongs to either the genus *Paronchocerca* or *Lemdana*. DNA sequences for these genera have not been deposited in the database as of the writing of this paper. While Lemdana species typically parasitize subcutaneous or organ connective tissue, *Paronchocerca* species occupy diverse sites in the avian host, including the circulatory system^14,26^. Additionally, the relatively short length of the microfilariae (approx. 100 µm) observed in peripheral blood aligns with the shorter microfilariae (≤ 200 µm in length) of the genus *Paronchocerca*, but distinguishes it from the middle or larger microfilariae (≥ 200 µm) of the genus *Lemdana*^14^. Taken together, our findings strongly support the conclusion that the filarioid detected in this study is a species of the genus *Paronchocerca*.

*Paronchocerca* is a member of the subfamily Splendidofilariinae with 21 described species, four of which are inquirendae^14,26^. Each species is seemingly restricted to a single avian family, and three species, *P. rousseloti*, *P. francolina*, and *P. badamii* (a species inquirenda), have been reported to parasitize the hearts of Phasianids^27–31^. Notably, the filarioid characterized in this study was distinct from these *Paronchocerca* parasites in host species, sampling localities, or microfilaria size (Supplementary Table S5), thus implying that the detected filarial parasite is likely to be a previously undescribed species. Further morphological and molecular studies focused on adult worms are necessary for taxonomic identification. Our results, particularly those regarding the site in the host and molecular data, will greatly contribute to future research.

Lesions and clinical signs have been documented in some filarioids that parasitize critical sites, such as the cardiovascular system, although avian filariasis is generally considered non-pathogenic^14^. Moderate inflammation was induced by *Splendidofilaria californiensis* in nodules on the luminal surface of the aorta, ultimately leading to a significant reduction in cardiac output^32^. Francolin birds that were heavily infected with the heartworm *P. rousseloti* exhibited easy fatigue when flying^28^. Sekiguchi et al. documented mild depression and open-mouth breathing in a hen infected with filarioids (presumably conspecific to the parasites in the present study) that was possibly due to collapsed air capillary regions in the lungs^15^. In this study, although we could not find any notable pathological changes in Case 1 with the exception of the occlusion of arteries in the lungs of Chabo3 due to endarteritis (Supplementary Fig. S2), the filarioids were possibly associated with the deaths of Chabos1–3, given the presence of microfilariae in multiple organs (Supplementary Fig. S6), the location of adult worms, and negative results for any pathogenic bacteria or viruses (Supplementary Table S1). Bacteria detected in each organ are likely a result of postmortem proliferation, given that the dead chickens were stored at high temperatures for over a day and no pathological changes from the bacteria were observed. In Case 2, vitamin A deficiency appeared to be the immediate cause of debilitation and death of the layers, as evidenced by typical signs of deficiency in the necropsied layers that included squamous metaplasia, periorbital edema, and accumulation of caseous material in the eyes^33^. However, it is crucial to note that previous studies have shown that poultry that are severely deficient in vitamin A display increased susceptibility to infections^34,35^, suggesting a possible exacerbation of debilitation in HLB1 by the filariasis. To address the potential burden of filarial parasites, studies evaluating their pathogenicity, including experimental infections, are required. Comparisons of parasite loads between deceased and healthy individuals could lead to an estimation of the health impact of filariasis.

Identification of vector species is critical for understanding the transmission dynamics of pathogens and developing preventive strategies for vector-borne diseases^36^. Previous studies have successfully identified vectors of avian filarioids such as *Cardiofilaria nilesi* and *Splendidofilaria* sp. through experimental ingestion and observation of infective larvae^14,37,38^. In this study, the nested PCR assay designed from the obtained mitogenome sequence was able to detect parasite DNA from the biting midge *C*. *arakawae*, suggesting that this hematophagous insect serves as a vector. *Culicoides arakawae* is distributed within a wide geographical range throughout East and Southeast Asia, including Japan, Taiwan, and Thailand^39–41^, and it is regarded as a vector of veterinary importance, as it can transmit the protozoan *Leucocytozoon caulleryi* that causes severe poultry disease^40^. Takaoka et al. reported that filarial larvae likely belonging to the subfamilies Lemdaninae and Splendidofilariinae were only detected in *C*. *arakawae* among eight examined *Culicoides* species in Japan, suggesting that this species also plays an important role in the transmission of avian filariae^42^. Control measures against *Culicoides* spp. such as the use of repellents/insecticide and physical control strategies during the seasonally active period of the midges are often applied to prevent viral or protozoan diseases^43^, and may be effective in the prevention of the filariasis. Nonetheless, it should be noted that the current study detected only filarial DNA in *C. arakawae* and that the presence of infective larvae in the midge needs to be confirmed to conclude the vector species of Onchocercidae sp. Other blood-sucking insects, including other species of *Culicoides* and black flies (Diptera: Simuliidae), may also be considered as potential vectors of filarial nematodes in the family Onchocercidae as reported elsewhere^44,45^.

To date, direct smearing and some concentration methods have been commonly used to detect filarial parasites in the peripheral blood of birds^15,24,46^. Although these techniques can reveal several key features, such as the presence or absence of a sheath and the shape of anterior/posterior extremity^14,25^, morphological identification of microfilariae remains challenging for non-experts due to the similarity and simplicity in their morphology. In this study, using the mitochondrial DNA information, we developed a nested PCR assay that successfully detected parasite DNA from biting midges, organs, and non-concentrated peripheral blood samples from live chickens. The resulting 426 bp-sequence was long enough to distinguish the parasite from other filarioids and exhibited the highest nucleotide identity of 91.08% with *Eufilaria sylviae* (data not shown). This nested PCR assay will be useful for further epidemiological studies such as those investigating the prevalence of the parasite in flocks throughout Japan, as well as the recent study using a TaqMan probe-based approach that revealed a widespread infection by *Splendidofilaria* sp. in Alaska grouse and ptarmigan species (Galliformes)^47^.

*Wolbachia* species are known as an important group of endosymbionts in filarial nematodes and arthropods and are classified into several lineages termed “supergroups”, where supergroups C, D, and F parasitize filarial hosts^48,49^. In this study, *Wolbachia* endosymbiont was detected in the DNA extracted from the lungs and was classified as supergroup F. Previous studies have indicated that *Wolbachia* spp. in filarial nematodes exhibit a stage-specific distribution in their host and play possible critical roles in the synthesis of metabolites and energy production^50,51^, and the depletion of the endosymbionts significantly disrupts its filarial host fitness and development^52^. As used for dirofilariasis and onchocerciasis^53,54^, the use of anti-*Wolbachia* drugs such as doxycycline and tetracycline might also serve as a control measure for filariasis caused by Onchocercidae sp. characterized in this study.

Since the first description of the genus *Pelecitus* by Railliet and Henry in 1910, a number of species across 16 genera within four subfamilies have been described as avian filarioids. These taxonomic classifications and species/genus relationships are predominantly based on the morphological characteristics of adult worms, as molecular approaches for phylogenetic classification remain limited^25,55^. Mitogenomic data encompassing protein-coding sequences and gene arrangements are robust tools for taxonomic revision. Previous studies using mitogenomic data have revealed new phylogenetic relationships among various parasites, including filarial nematodes. These findings provide insights into traditional taxonomy based on morphology, ecology, and partial nuclear/mitochondrial gene sequences, ultimately leading to the discovery of cryptic species and new taxonomic classifications^20,56–58^. A comprehensive taxonomic revision would be necessary for avian filarioids using mitogenomes in combination with other critical features such as morphology. The two primer sets employed in this study (ND5F & ND5R and Cytb_F & Cytb_R) that were designed from the alignment of mitochondrial sequences of members of the family Onchocercidae are theoretically applicable to diverse groups of filarioids. This approach, which facilitates mitogenome sequencing, is anticipated to contribute significantly to the expansion of the existing database, ultimately advancing a deeper understanding of the systematic classification of avian filarioids.

## Materials and methods

### Ethical approval and consent to participate

All the animal experiments were carried out under the guidance of Institute for Laboratory Animal Research, which was based on Fundamental Guidelines for Proper Conduct of Animal Experiment and Related Activities in Academic Research Institutions under the jurisdiction of the Ministry of Education, Culture, Sports, Science and Technology, Japan. The procedures were approved by the Animal Experiment Committee of the Graduate School of Veterinary Medicine, Hokkaido University (Sapporo, Japan). This study is reported in compliance with the ARRIVE (Animals in Research: Reporting In Vivo Experiments) guidelines.

### Material collection

Specimens were collected from six chickens (designated as Chabos1–3, HLB1, AR1, and CB1) that were raised in two different backyard chicken flocks in Hiroshima Prefecture, Japan. Detailed case histories are presented in the previous section. We also collected blood specimens from seven Chabos that were housed together with Chabos1–3 and HLB1, and we then performed microfilaria concentration experiments using acetone described below.

### Histopathological examination

The collected organs were fixed in neutral-buffered 10% formalin solution and subjected to routine histologic processing. Samples were embedded in paraffin, sectioned at 3 µm, and stained with hematoxylin and eosin (H&E).

### Microfilaria detection in blood samples

We employed a microfilaria concentration method using acetone described by Ohishi et al., with slight modifications^59^. Initially, an acetone solution was prepared by mixing 5 ml of filtered 0.5% methylene blue, 5 ml of acetone, 0.2 g of sodium citrate, and 90 ml of distilled water. Then we added 100 µl of blood sample to 900 µl of the solution, followed by a centrifugation step at 1500 rpm for 10 min. After centrifugation, the supernatant was carefully discarded, retaining 0.5 ml of the solution at the bottom. The sediment was then thoroughly washed with 10 ml of distilled water and the suspension was centrifuged once again at 1500 rpm for 10 min. We discarded the supernatant gently until its volume equaled that of the bottom sediment, and then mixed the sediment thoroughly with the remaining supernatant. The resulting solution was sealed in a cover glass and subsequently examined under a microscope.

### Bacteriological/virological examinations

Multiple organs, including the brain, heart, kidney, liver, spleen, and lung, from the chickens were homogenized and used for bacterial tests (Supplementary Table S1). The resulting homogenates were plated onto 5% sheep blood and DHL agar plates. The sheep blood agar plates were incubated under anaerobic conditions at 37 °C for 48 h, while the DHL agar plates were incubated under aerobic conditions at 37 °C for 24 h. Bacteria were identified using commercial kits (Rapid ID 32 E, ID 32 Staph, API 20 E, and API 50 CH) purchased from Japan bioMérieux (Tokyo, Japan).

Tracheal and cloacal swabs from Chabos1–3 were subjected to the ESPLINE® Influenza A & B-N assay (Fujirebio, Tokyo, Japan). Homogenates of brains and livers, each at a volume of 200 µl, were inoculated into embryonated eggs (aged 9–10 days) for virus isolation, and the viability of these eggs was assessed at 24 and 48 h post-inoculation. The allantoic fluid collected at 48 h after inoculation was subsequently used in a hemagglutination assay to detect influenza A and Newcastle disease viruses. To detect other pathogenic viruses, we inoculated the homogenates as described above and observed whether the embryos exhibited curling or dwarfing after five days.

### DNA extraction, PCR, and Sanger sequencing

Total DNA was extracted from the organ homogenates (lung, liver, spleen, kidney, and heart) of the four necropsied chickens (Chabos1–3 and HLB1), and from the blood samples collected from seven Chabos using the DNeasy® Blood and Tissue kit (QIAGEN) following the manufacturer’s protocol. PCR was performed using a primer set specific for the 18S rRNA gene (rDNA) (988F and 1912R)^60^ and another set specific for 28S rRNA gene (Nematode1 and Nematode2)^61^. The cycling conditions are listed in Supplementary Table S2. The PCR products were purified using the NucleoSpin® Gel and PCR Clean-up kit (Macherey-Nagel, Düren, Germany) or the ExoSAP-IT PCR Product Cleanup Reagent (Thermo Fisher Scientific, Waltham, MA, USA). The purified products were sequenced by primer walking using a BigDye Terminator v3.1 Cycle Sequencing Kit (Applied Biosystems, Foster City, CA, USA).

### Mitochondrial genome (mitogenome) construction

To sequence the mitochondrial cytochrome *c* oxidase I (*COI*) gene that is the most commonly sequenced gene from filarial parasites and has been deposited in the sequence database, we performed PCR with COIintF and COIintR primers targeting the partial *COI* gene of the Onchocercidae family^62^. However, this reaction was unsuccessful for any DNA extracted from tissues (data not shown), and this is consistent with a previous report by Sekiguchi et al.^15^. Alternatively, an amplicon-based mitogenome construction was performed. A schematic representation of the PCR strategy is shown in Supplementary Fig. S3. Primer sets and PCR conditions used in this study are listed in Supplementary Table S2. Initially, we amplified and sequenced partial cytochrome *b* (781 bp) and NADH dehydrogenase subunit 5 (505 bp) using two primer sets. These primer sets were designed by aligning the mitochondrial sequences of five species of the family Onchocercidae, including *Brugia malayi* (MT149211), *Wuchereria bancrofti* (AP017705), *Loa loa* (HQ186250), *Dirofilaria repens* (KX265049), and *Acanthocheilonema viteae* (HQ186249). We designed new primer sets for the sequences (FiMtG_ND5F and FiMtG_CytbR, FiMtG_CytbF and FiMtG_ND5R) and amplified the mitogenome using DNA extracted from the lungs of Chabo2 in two overlapping PCRs. The PCR products were analyzed by electrophoresis, and this was followed by purification with the NucleoSpin® Gel and PCR Clean-up kit. An Illumina sequencing library was generated from the purified amplicons using the Nextera XT DNA Library Preparation Kit (Illumina, Hayward, CA, USA). The Illumina MiSeq platform was used for sequencing with the MiSeq Reagent Kit v3 for 600 cycles. Sequencing reads were assembled using the CLC Genomics Workbench v20.0.4 (Qiagen, Hilden, Germany), generating two contigs of 6527 and 6943 bp. These contigs were manually connected by eliminating overlapping ends, resulting in a linear sequence of 13,380 bp.

The circular mitogenome was not completed by de novo assembly due to the AT-rich region between *COIII* and tRNA-Ala (Supplementary Fig. S3), which is common in other filarial nematode mitogenomes. TA cloning was performed to fill this gap. Briefly, primers (Gap1F and Gap1R) were designed for both ends of the linear sequence, and PCR was performed using KOD Plus Neo (Toyobo, Osaka, Japan). The PCR product was analyzed and purified as described above, and it was ligated to the pMD20-T-vector (Takara Bio Inc.) using a 10 × A-attachment mix (Toyobo) and Ligation Mighty Mix (Takara Bio Inc.). DH5α cells were employed for transformation, and the cells were cultured on Luria–Bertani (LB) Agar medium (Invitrogen, Carlsbad, CA, USA). The single colony was further used for PCR and Sanger sequencing with the M13 M4 and M13 RV primers.

The constructed mitogenome was annotated using the MITOS web server (http://mitos.bioinf.uni-leipzig.de/index.py)^63^ with the Invertebrate mitochondrial code (translation table 5). The curation of the predicted genes was performed by aligning the nucleotide sequences with those of other filarial nematodes using MAFFT v7.450^64^.

### Phylogenetic analysis

Nuclear 18S rRNA, 28S rRNA, and mitochondrial *COI* gene sequences were used for phylogenetic analysis. The ascarid nematodes and *Thelazia callipaeda* (family: Thelaziidae) were selected as outgroups based on previous studies^15,20,25^. Alignments of nucleotide sequences were performed using MAFFT v7.450^64^, and the best-fit models were estimated according to the Bayesian information criterion of Kakusan4^65^ for the subsequent analysis based on Bayesian inference. The K80 model ^66^ with a gamma distribution was selected for 18S rRNA gene, and the general time-reversible (GTR) model^67^ with a gamma distribution was selected for 28S rRNA gene. A codon-proportional model was selected for *COI* gene. HKY85^68^ with a gamma distribution was used for the first and third codon position, and GTR with a gamma distribution was used for the second position. The Bayesian tree was estimated using MrBayes v3.2.6^69^ with 5,000,000 generations via the Markov Chain Monte Carlo algorithm with a sample frequency of 500 and a burn-in of 500,000. Assessments of the convergence and sample size adequacy for each parameter were performed using Tracer v1.6^70^. Two independent runs were summarized to reconstruct the 50% majority-rule consensus tree and to estimate the posterior probability of each node. The generated trees are presented in FigTree v1.4.3.

### PCR-based assay for detection of potential vectors

In Case 1, a commercial light trap was attached adjacent to the hen house overnight to capture midges. A total of 50 midges that were morphologically identified as *Culicoides* spp. upon microscopic examination were used for the analysis. After washing three times with phosphate-buffered saline (PBS), each midge was incubated in 0.1% sodium hypochlorite solution for 1 min at room temperature to minimize the risk of DNA contamination. The remaining hypochlorite was removed by two successive washes with PBS. DNA extraction from midge samples was performed using the NucleoSpin® DNA insect kit (Macherey–Nagel) according to the manufacturer’s protocol, yielding a DNA solution of 30 µl. First, we performed PCR and Sanger sequencing targeting the mitochondrial *COI* of *Culicoides arakawae* for the species identification of midges. After molecular identification, a nested PCR assay designed in this study was employed to detect the filarial *COI* sequence from the extracted DNA. Briefly, the first PCR was conducted with the primers Oncho_CO1F and Oncho_CO1R (Supplementary Table S2) using 1 µl of extracted DNA. Following tenfold dilutions of the first PCR products, 1 µl of diluted product was used for the second step PCR with the primers Oncho_CO1FN and Oncho_CO1RN (Supplementary Table S2). The midges were assessed using the CulicoidesA_CO1F and CulicoidesA_CO1R primers. The PCR products were purified and sequenced using the BigDye Terminator v3.1 Cycle Sequencing Kit as described above.

### Detection and characterization of a *Wolbachia* endosymbiont

PCR-based detection of *Wolbachia*, a common endosymbiont of filarial parasites, was conducted on the DNA extracted from the lungs of Chabo2. The partial sequence of *Wolbachia* 16S rDNA was amplified using the primer pair EHR16SD and 1513R^71,72^. After purification of the PCR product, the full-length sequence was determined using primer walking. Bayesian phylogenetic inference was performed using the K80 model with gamma distribution as described above.

## Conclusion

In this study, we used histological and genetic analyses to characterize an avian filarioid that affects domestic chickens in Hiroshima Prefecture, Japan. These results provide several important insights into the nature of this filarial parasite. First, the parasite was most likely a previously undescribed species of the genus *Paronchocerca*. Second, filariasis caused by the parasite may be prevalent in a wide area of Japan, with occasional clinical manifestations or death. Third, *C*. *arakawae*, a widely distributed midge in East Asia, is a potential vector of this parasite. The results of this study would contribute to a deeper understanding of the prevalence, geographical distribution, and detailed life cycle, all of which are prerequisites for proper health management measures in poultry flocks. Furthermore, it is necessary to evaluate the prevalence and health burden of this neglected avian filariasis in other regions worldwide.

### Supplementary Information

Supplementary Information.

## Data Availability

The sequences analyzed in this study are available under the accession numbers provided in Results section. Any additional information related to this study is available from the corresponding authors upon request.
