# Supplementary material for: A filarial parasite potentially associated with the health burden on domestic chickens in Japan
Source: Sci Rep. 2024 Mar 15;14:6316. doi: 10.1038/s41598-024-55284-2 (PMC10943242; doi:10.1038/s41598-024-55284-2)
Supplement: Supplementary file 1 — Supplementary Information. [file 41598_2024_55284_MOESM1_ESM.docx]

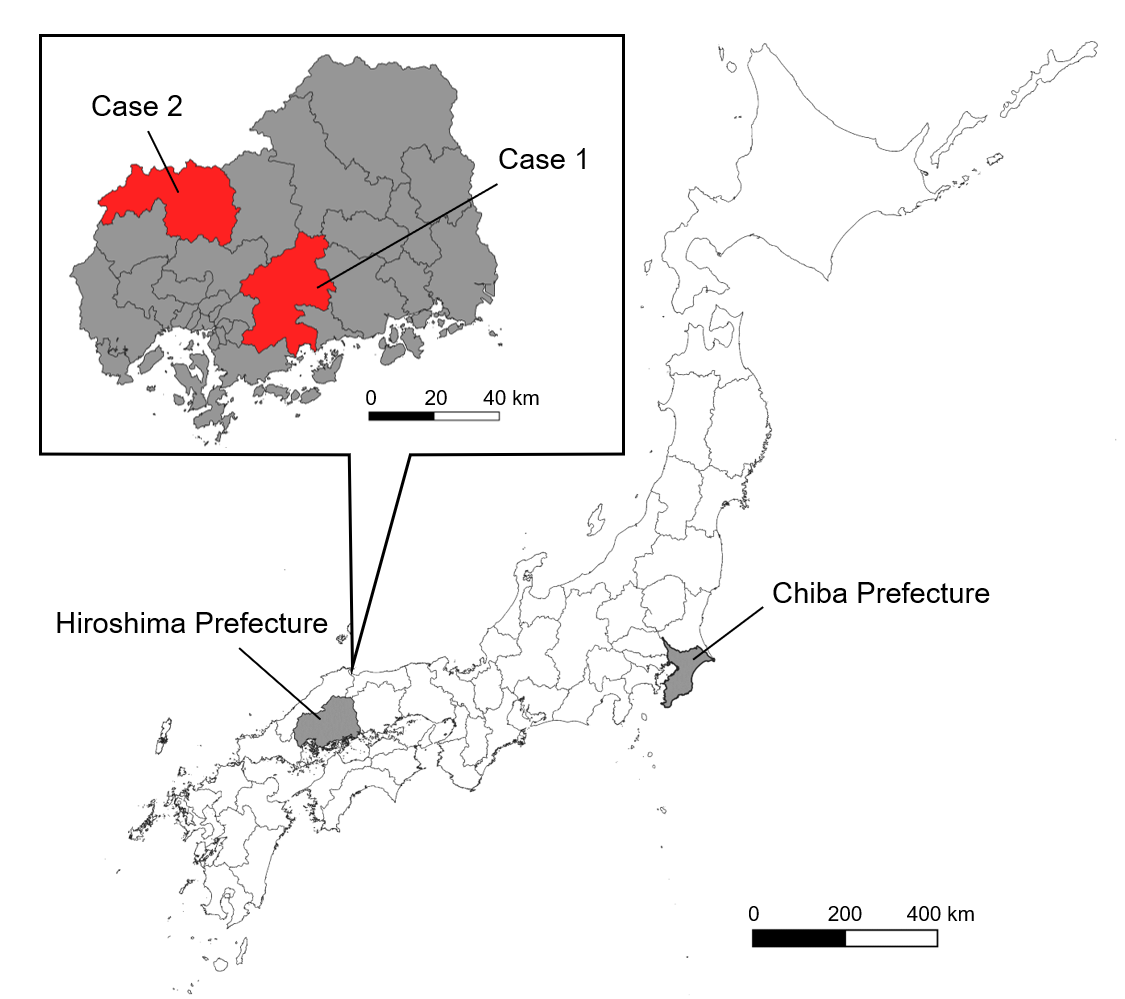


**Supplementary Fig. S1. A map showing the locations where Onchocercidae sp. was detected in domestic chickens.** The two prefectures, Hiroshima and Chiba, where Onchocercidae sp. was detected, are highlighted in gray. The inset shows the municipalities in Hiroshima Prefecture where Case 1 and Case 2 in this study were identified. The map was created by QGIS v3.16 (available at <https://www.qgis.org/ja/site/forusers/download.html>) using vector map data from National Land Information Division, National Spatial Planning and Regional Policy Bureau, MLIT of Japan (<https://nlftp.mlit.go.jp/ksj/index.html>).


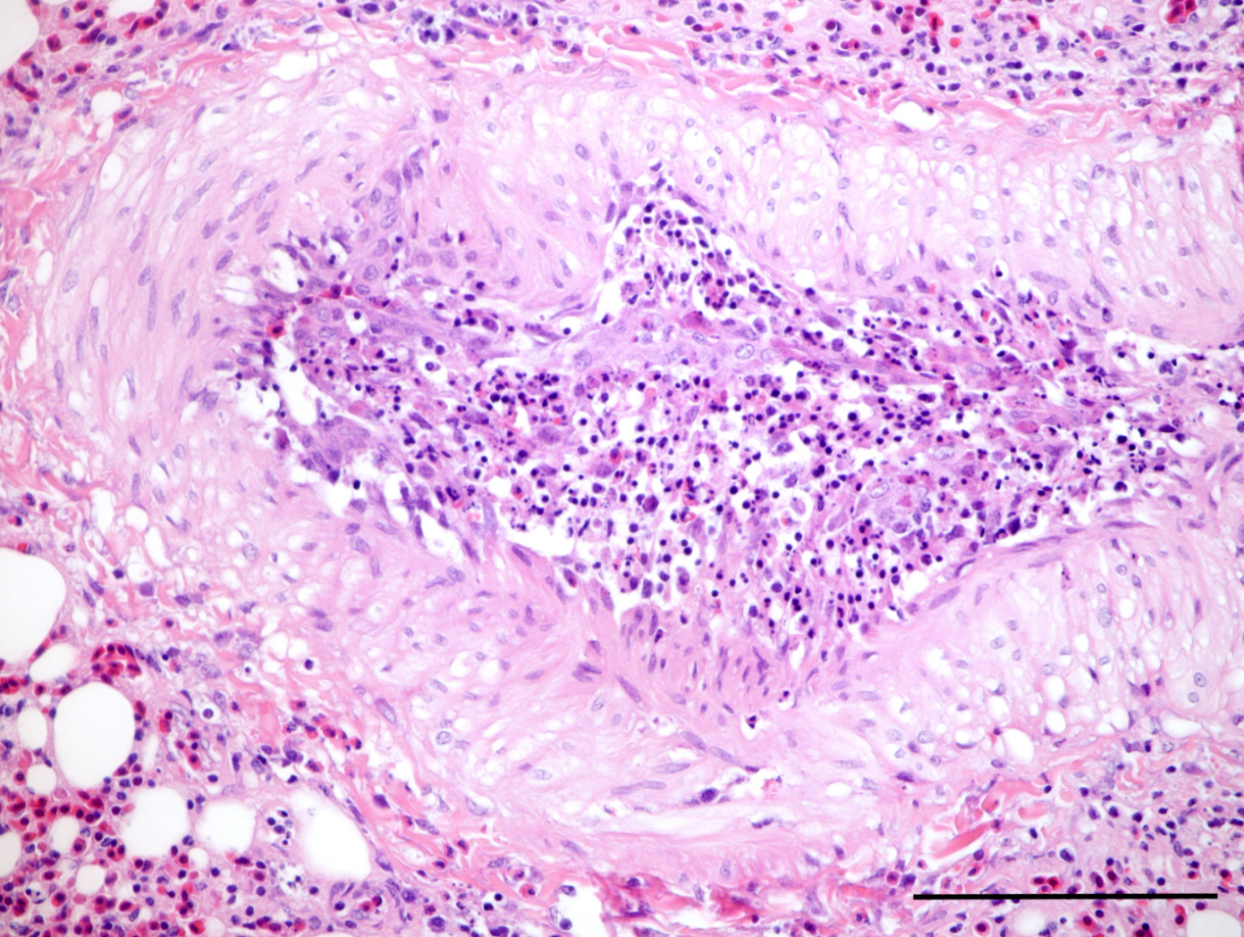


**Supplementary Fig. S2. A microscopic image showing the obliterating endarteritis and vacuolation of the smooth muscle wall of the medium-sized artery in the lungs of a Chabo.** The section was stained with H&E. Scale bar = 100 µm


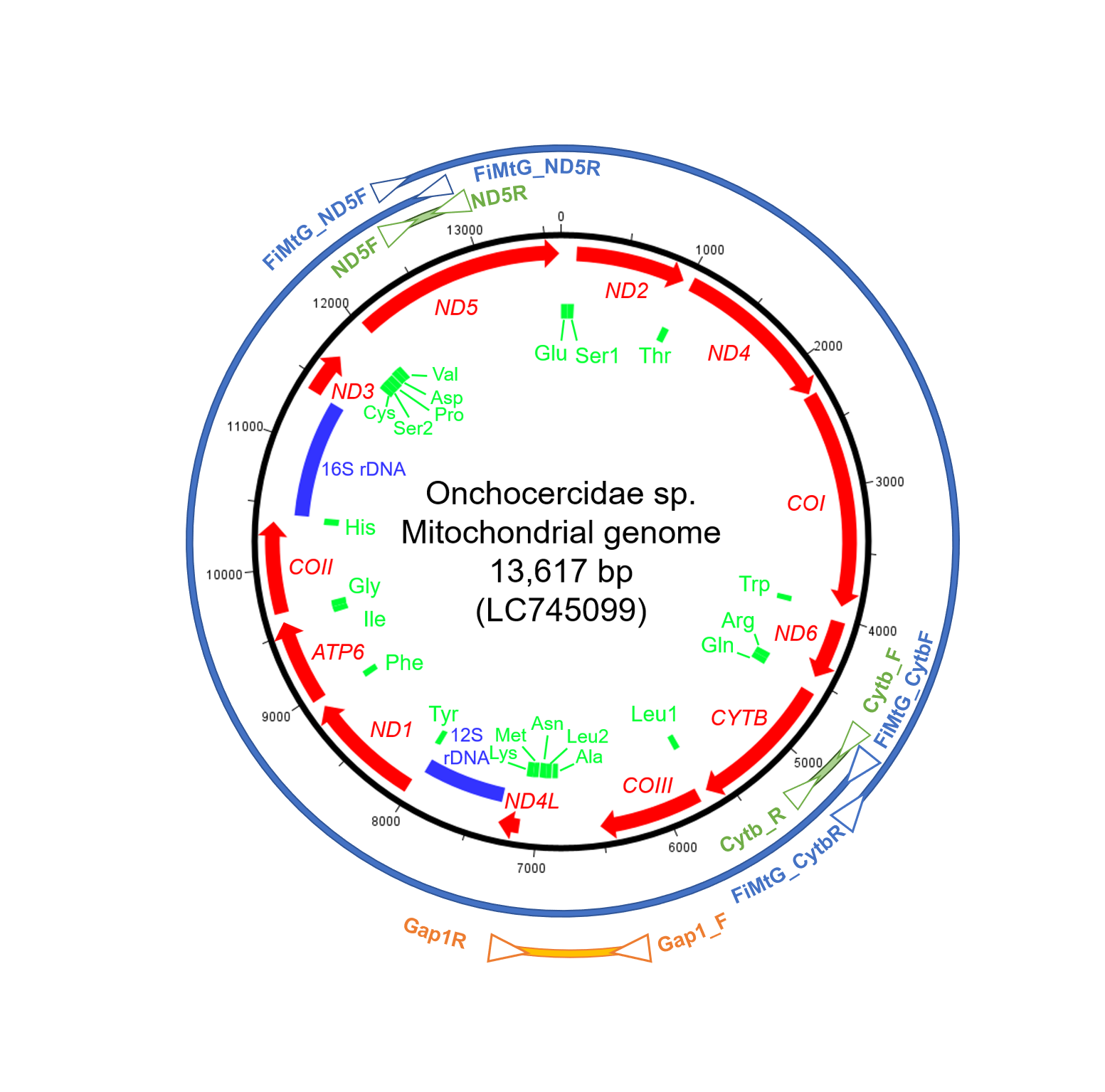


**Supplementary Fig. S3.** **Illustration of amplicon-based mitogenome construction.** Arrowheads indicate the primer positions.


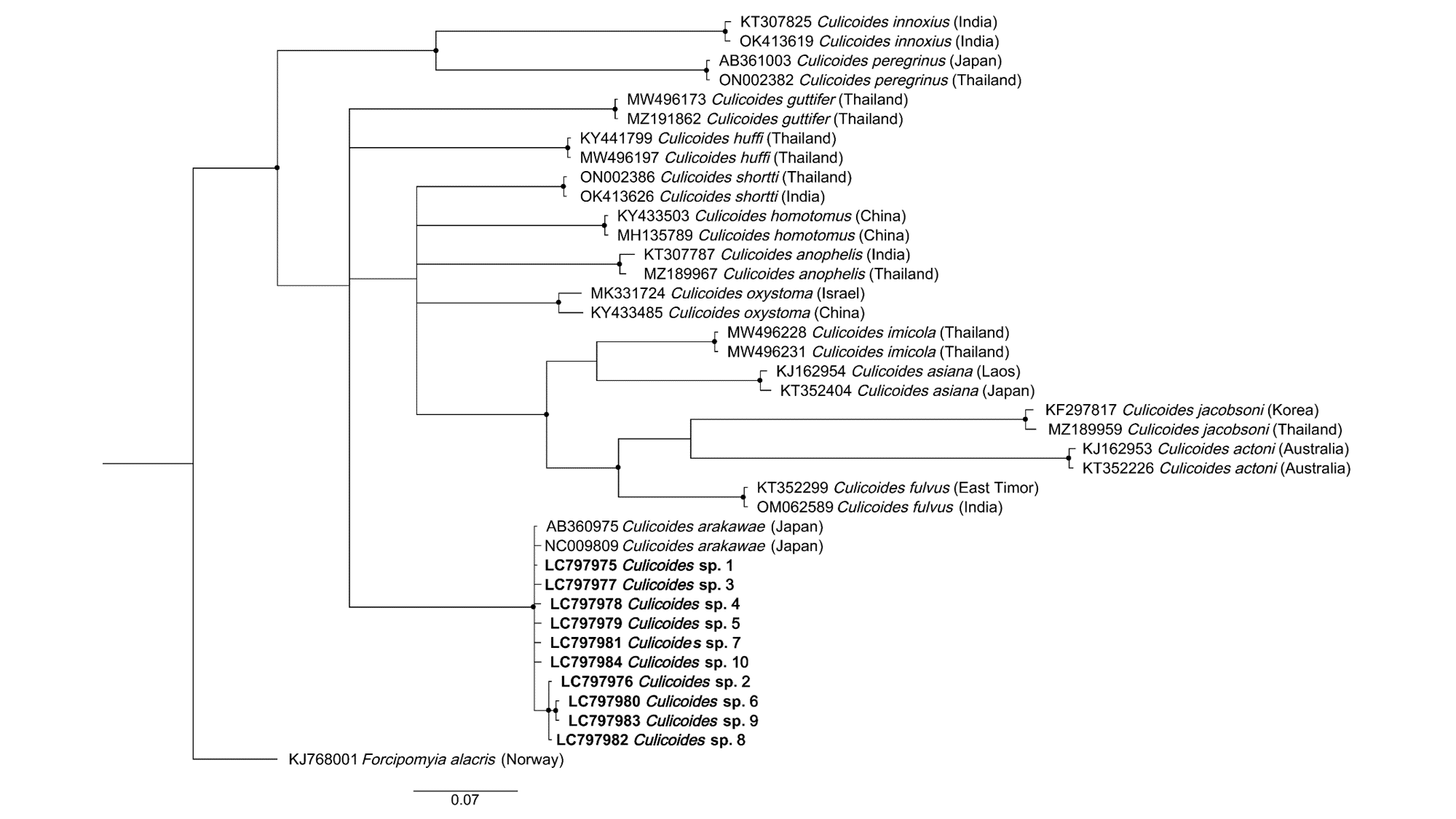


**Supplementary Fig. S4. Bayesian phylogenetic tree constructed using the mitochondrial *COI* gene sequences of *Culicoides* spp.** The samples of midges obtained in this study were designated as *Culicoides* sp. 1–10. Black dots indicate nodes with posterior probability of >0.75.


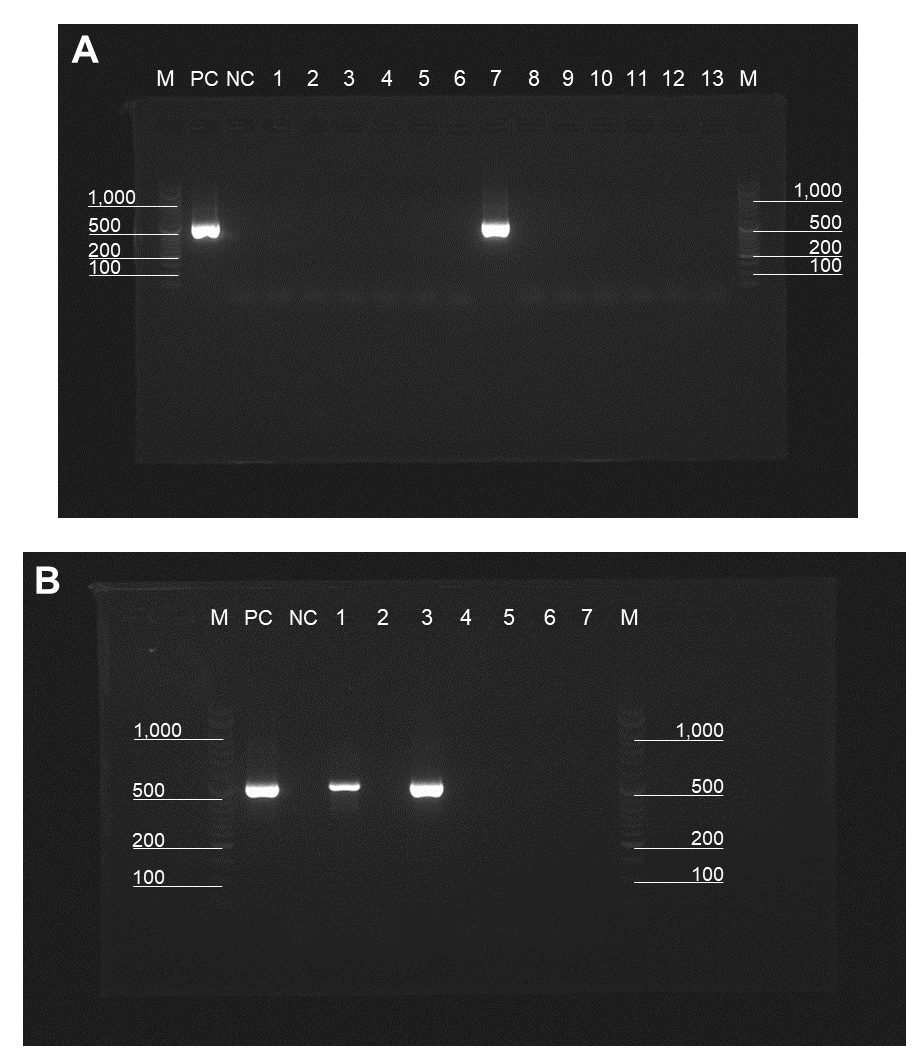


**Supplementary Fig. S5. Full length gel images of** **molecular detection of Onchocercidae sp. DNA from (A) *Culicoides arakawae* and (B) blood of Chabos that were housed with the dead Chabos.** The original gel images of Figure 5 are presented. The PCR products were analyzed in 2.0% agarose gels. (A) Lane M, DNA ladder; lane PC, positive control; lane NC, negative control; lanes 1–13, DNA extracted from *C*. *arakawae*. (B) Lanes 1–7, DNA extracted from the blood samples of Chabos.


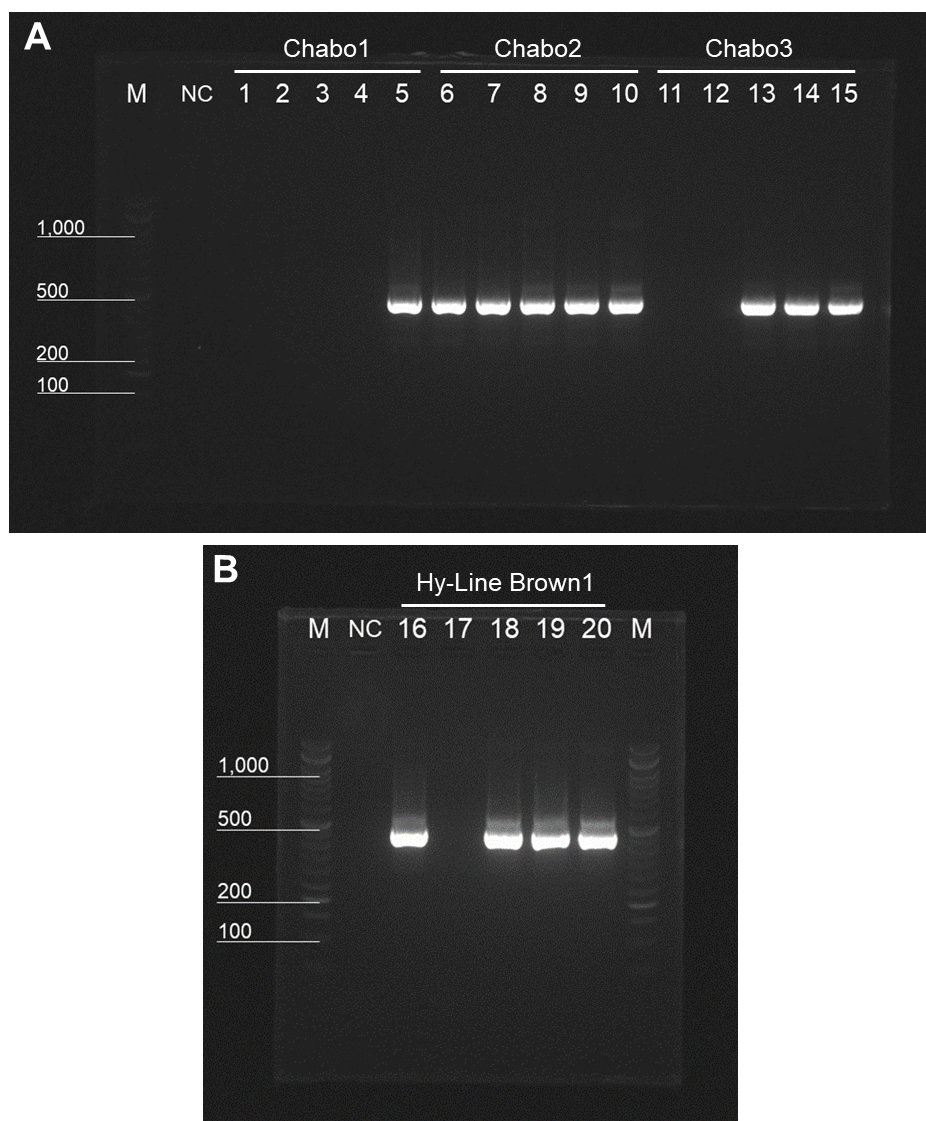


**Supplementary Fig. S6.** **Molecular detection of Onchocercidae sp. DNA from tissues of (A) Chabos1–3 and (B) Hy-Line Brown1 using the nested-PCR assay.** The PCR products were analyzed in a 2.0% agarose gel. Lane M, DNA ladder; lane NC, negative control; lanes 1, 6, 11,16, liver DNA; lanes 2,7,12,17, spleen DNA; lanes 3, 8, 13,18 kidney DNA; lanes 4, 9, 14,19, heart DNA; lanes 5, 10, 15, 20, lung DNA.

| Supplementary Table S1. Bacterial isolation from tissue homogenates | | | | | |
| --- | --- | --- | --- | --- | --- |
|  | Specimen | Tissue | Isolated bacteria | Plate | Colony forming unit/gram |
| Case 1 | Chabo1 | Brain | *Escherichia coli* | DHL agar | 1.0×10^4^< |
|  |  |  | *Klebsiella pneumoniae* | DHL agar | 2.2×10^2^ |
|  |  | Kidney | *Escherichia coli* | DHL agar | 1.0×10^4^< |
|  |  |  | *Klebsiella pneumoniae* | DHL agar | 6.0×10 |
|  |  | Spleen | *Escherichia coli* | DHL agar | 1.0×10^4^< |
|  |  | Lung | *Escherichia coli* | DHL agar | 1.5×10^3^ |
|  | Chabo2 | Brain | *Klebsiella pneumoniae* | DHL agar | 1.0×10^4^< |
|  |  | Heart | *Klebsiella pneumoniae* | DHL agar | 1.0×10^4^< |
|  |  | Kidney | *Klebsiella pneumoniae* | DHL agar | 1.0×10^4^< |
|  |  | Liver | *Klebsiella pneumoniae* | DHL agar | 7.5×10^2^ |
|  |  | Spleen | *Klebsiella pneumoniae* | DHL agar | 1.7×10^2^ |
|  |  | Lung | *Bacillus cereus* | 5% sheep blood agar | 1.0×10^4^ |
|  | Chabo3 | Brain | *Escherichia coli* | DHL agar | 1.0×10^4^< |
|  |  | Kidney | *Bacillus cereus* | 5% sheep blood agar | 1.0×10^4^< |
|  |  | Liver | *Staphylococcus hyicus* | 5% sheep blood agar | 1.3×10^3^ |
|  |  | Spleen | *Staphylococcus hyicus* | 5% sheep blood agar | 5.2×10^3^ |
|  |  | Lung | *Bacillus cereus* | 5% sheep blood agar | 4.7×10^5^ |
| Case 2 | HLB1 | Eyelid | *Staphylococcus schleiferi*^a^ | 5% sheep blood agar | 1.4×10^4^ |
|  | AR1 | Eyelid | *Staphylococcus schleiferi*^a^ | 5% sheep blood agar | 3.0×10^4^ |
|  |  |  | *Pasteurella* sp.^b^ | 5% sheep blood agar | 5.0×10^3^ |
|  |  | Submandibular gland | *Pasteurella* sp.^b^ | 5% sheep blood agar | 6.0×10^5^ |
|  |  | Lung | *Staphylococcus schleiferi*^a^ | 5% sheep blood agar | 1.2×10^6^ |
|  |  |  | *Pasteurella* sp.^b^ | 5% sheep blood agar | 3.0×10^5^ |
|  |  |  | *Escherichia coli* | DHL agar | 3.0×10^6^ |
|  |  | Liver | *Staphylococcus schleiferi*^a^ | 5% sheep blood agar | 2.5×10^3^ |
|  |  |  | *Escherichia coli* | DHL agar | 7.0×10^3^ |
|  | CB1 | Eyelid | *Staphylococcus schleiferi*^a^ | 5% sheep blood agar | 2.5×10^3^ |
|  |  | Heart | *Staphylococcus schleiferi*^a^ | 5% sheep blood agar | 4.0×10^3^ |
| ^a^ A commensal bacterium that exists on the surface of animals and can sometimes cause opportunistic infections. | | | | | |
| ^b^ Species-specific PCR confirmed that this bacterium was not *Pasteurella multocida*. | | | |  |  |

| Supplementary Table S2. Primer sequences and PCR conditions | | | |  |  |  |
| --- | --- | --- | --- | --- | --- | --- |
| Primer name | Target (organism) | Target (gene) | Primer sequence (5'→3') | Product size (bp) | PCR enzyme | Cycling conditions |
| 988F | Onchocercidae sp. | Nuclear 18S rDNA | CTCAAAGATTAAGCCATGC | 998 | Tks Gflex (Takara Bio Inc., Shiga, Japan) | 94 °C 60 s, 40 cycles of 98 °C 10 s, 55 °C 15 s, 68 °C 30 s |
| 1912R |  |  | TTTACGGTCAGAACTAGGG |  |  |  |
| Nematode1 | Onchocercidae sp. | Nuclear 28S rDNA | GCGGAGGAAAAGAAACTAA | 855 | Tks Gflex | 94 °C 60 s, 40 cycles of 98 °C 10 s, 55 °C 15 s, 68 °C 30 s |
| Nematode2 |  |  | ATCCGTGTTTCAAGACGGG |  |  |  |
| ND5F | Onchocercidae sp. | Mitochondrial *ND5* | GGKCAGTATCCTTTTGGTAG | 505 | Tks Gflex | 94 °C 60 s, 40 cycles of 98 °C 10 s, 55 °C 15 s, 68 °C 30 s |
| ND5R |  |  | CCTTACTACAAYAMCCACTAG |  |  |  |
| Cytb_F | Onchocercidae sp. | Mitochondrial *CYTB* | TKCAYTCTAAKGGTGCTTC | 781 | Tks Gflex | 94 °C 60 s, 40 cycles of 98 °C 10 s, 55 °C 15 s, 68 °C 30 s |
| Cytb_R |  |  | GGATAATCWGTRGGATAATG |  |  |  |
| FiMtG_ND5F | Onchocercidae sp. | Mitogenome | GTTTGCTCTTTGGTTGAAGAAGATGC | 6,238 | PrimeSTAR GXL DNA polymerase (Takara Bio Inc.) | 45 cycles of 98 °C 10 s, 68 °C 6 min |
| FiMtG_CytbR |  |  | CCATTCAGGAACAACATGTGCAGGTC |  |  |  |
| FiMtG_ND5R | Onchocercidae sp. | Mitogenome | GAATAGCCACGATAATCTTGATGACC | 8,082 | PrimeSTAR GXL DNA polymerase | 45 cycles of 98 °C 10 s, 60 °C 15 s, 68 °C 8 min |
| FiMtG_CytbF |  |  | GTCTTACTGGTGTTTGGTTAAGAGGTG |  |  |  |
| Gap1F | Onchocercidae sp. | Mitochondrial  non-coding region | TATGCAAGCTTTTGACATGTC | 573 | KOD Plus Neo (Toyobo, Osaka, Japan) | 94 °C 2 min, 42 cycles of 98 °C 10 s, 60 °C 30 s, 68 °C 45 s |
| Gap1R |  |  | CACCCACTAATCAACAATTAGC |  |  |  |
| M13 M4 | Onchocercidae sp. | T-Vector pMD20 cloning site | GTTTTCCCAGTCACGAC | 748 | EmeraldAmp PCR Master Mix (Takara Bio Inc.) | 30 cycles of 98 °C 10 s, 60 °C 30 s, 72 °C 90 s |
| M13 RV |  |  | CAGGAAACAGCTATGAC |  |  |  |
| EHR16SD | *Wolbachia* | 16S rDNA | GGTACCYACAGAAGAAGTCC | 1,030 | Tks Gflex | 94 °C 60 s, 45 cycles of 98 °C 10 s, 55 °C 15 s, 68 °C 30 s |
| 1513R |  |  | ACGGYTACCTTGTTACGACTT |  |  |  |
| Oncho_CO1F | Onchocercidae sp. | Mitochondrial *COI* | CTATTTTGATCGGTGGCTTCG | 849 | Tks Gflex | 94 °C 60 s, 45 cycles of 98 °C 10 s, 60 °C 15 s, 68 °C 30 s |
| Oncho_CO1R |  |  | TTCCACTCAAACCACCTACAG |  |  |  |
| Oncho_CO1FN | Onchocercidae sp. | Mitochondrial *COI* (for nested PCR) | AGTGGAGGGTCAACCTGAGATA | 470 | Tks Gflex | 94 °C 60 s, 35 cycles of 98 °C 10 s, 60 °C 15 s, 68 °C 30 s |
| Oncho_CO1RN |  |  | GATGACCCCAAACAGAAGTACC |  |  |  |
| CulicoidesA_CO1F | *Culicoides arakawae* | Mitochondrial *COI* | TTAGGAGCCCCCGATATAGCTT | 475 | Tks Gflex | 94 °C 60 s, 45 cycles of 98 °C 10 s, 60 °C 15 s, 68 °C 30 s |
| CulicoidesA_CO1R |  |  | TATAAACTTCGGGGTGGCCAAA |  |  |  |

| Supplementary Table S3. Annotated mitochondrial genes of Onchocercidae sp. | | | | |  |
| --- | --- | --- | --- | --- | --- |
| Gene name | Position | Size (bp) | Number of amino acids | Init./term. codon | Intergenic nucleotides |
| tRNA-Glu | 1–57 | 57 |  |  | 0 |
| tRNA-Ser1 | 58–114 | 57 |  |  | 3 |
| *ND2* | 118–957 | 840 | 280 | TTG/TAG | 0 |
| tRNA-Thr | 958–1,020 | 63 |  |  | −1 |
| *ND4* | 1,020–2,249 | 1,230 | 410 | ATG/TAA | 7 |
| *COI* | 2,257–3,906 | 1,650 | 550 | GTT*/TAG | 2 |
| tRNA-Trp | 3,909–3,966 | 58 |  |  | 54 |
| *ND6* | 4,021–4,473 | 453 | 151 | TAT*/TAA | −2 |
| tRNA-Arg | 4,472–4,525 | 54 |  |  | 3 |
| tRNA-Gln | 4,529–4,582 | 54 |  |  | 0 |
| *CYTB* | 4,583–5,671 | 1,089 | 363 | ATT/TAA | 7 |
| tRNA-Leu1 | 5,679–5,736 | 58 |  |  | 0 |
| *COIII* | 5,737–6,516 | 780 | 260 | ATT/TAG | 318 |
| tRNA-Ala | 6,835–6,890 | 56 |  |  | 3 |
| tRNA-Leu2 | 6,894–6,947 | 54 |  |  | 0 |
| tRNA-Asn | 6,948–7,005 | 58 |  |  | 7 |
| tRNA-Met | 7,013–7,067 | 55 |  |  | 2 |
| tRNA-Lys | 7,070–7,125 | 56 |  |  | 1 |
| *ND4L* | 7,127–7,289 | 163 | 55 | GTA*/T** | 4 |
| 12S rDNA | 7,294–7,967 | 674 |  |  | 1 |
| tRNA-Tyr | 7,969–8,022 | 54 |  |  | −3 |
| *ND1* | 8,020–8,896 | 877 | 293 | TTG/T** | 0 |
| tRNA-Phe | 8,897–8,955 | 59 |  |  | 0 |
| *ATP6* | 8,956–9,540 | 585 | 195 | ATT/TAA | 9 |
| tRNA-Ile | 9,550–9,608 | 59 |  |  | 0 |
| tRNA-Gly | 9,609–9,662 | 54 |  |  | 3 |
| *COII* | 9,666–10,364 | 699 | 233 | ATT/TAA | −2 |
| tRNA-His | 10,363–10,418 | 56 |  |  | 0 |
| 16S rDNA | 10,419–11,388 | 970 |  |  | 2 |
| *ND3* | 11,391–11,727 | 337 | 113 | CTT*/T** | 0 |
| tRNA-Cys | 11,728–11,784 | 57 |  |  | 0 |
| tRNA-Ser2 | 11,785–11,839 | 55 |  |  | 0 |
| tRNA-Pro | 11,840–11,898 | 59 |  |  | 0 |
| tRNA-Asp | 11,899–11,956 | 58 |  |  | 0 |
| tRNA-Val | 11,957–12,012 | 56 |  |  | 0 |
| *ND5* | 12,013–13,608 | 1,596 | 532 | TTT/TAG | 9 |

*Start codons are tentative and not experimentally identified.

**TAA stop codon is presumably completed by the addition of 3' A residues to the mRNA.

Supplementary Table S4. Nucleotide identity (%) in pairwise comparisons between mitochondrial cytochrome *c* oxidase subunit I (*COI*) of species of the family Onchocercidae

The font color represents the host of the parasite, where orange indicates that the parasite has avian hosts, blue indicates mammalian hosts, and green indicates reptilian hosts.

| Supplementary Table S5. Comparison of *Paronchocerca* spp. detected from the heart of Phasianids | | | | |
| --- | --- | --- | --- | --- |
| Species | Reported host species (country) | Site in the host | Microfilariae length×width (µm) | Reference |
| *P*. *rousseloti* | *Scleroptila finschi* (Congo), *Campocolinus coqui* (Congo),  *Pternistis leucoscepus* (Somalia), *Francolinus francolinus* (Russia and India), *Francolinus pondicerianus* (India) | Right auricle of heart, heart cavity,  pulmonary arteries, subcutaneous tissue of head and neck | 125 µm in length (in the uterus, the width was not described) | Chabaud and Biocca, 1951; Kasimov, 1952; Bump and Bump, 1964 |
|  |  |  |  |  |
|  |  |  |  |  |
| *P*. *francolina* | *Francolinus pondicerianus* (India) | Lung, heart, air sacs | 151–165×5 | Jairajpuri and Siddiqi, 1970 |
| *P*. *badamii* (species inquirenda) | *Gallus gallus domesticus* (India) | Heart | 20–24×2.2–4 | Bhalerao and Rao, 1944 |
| Onchocercidae sp. | *Gallus gallus domesticus* (Japan) | Heart | 87–110×4–7 | This study |
